# Supplementary material for: Survival and development of potato psyllid (Hemiptera: Triozidae) on Convolvulaceae: Effects of a plant-fungus symbiosis (Periglandula)
Source: PLoS One. 2018 Sep 11;13(9):e0201506. doi: 10.1371/journal.pone.0201506 (PMC6133269; doi:10.1371/journal.pone.0201506)
Supplement: S1 Table — (DOCX) [file pone.0201506.s001.docx]

| Species | Distance from Potato |
| --- | --- |
| *Convolvulus equitans* | 0.308 |
| *Calystegia silvatica* | 0.303 |
| *Turbina corymbosa* | 0.301 |
| *Ipomoea pandurata* | 0.287 |
| *Ipomoea imperati* | 0.295 |
| *Ipomoea leptophylla* | 0.292 |
| *Ipomoea tricolor* | 0.287 |
| Potato |  |
| *Convolvulus tricolor* | 0.312 |
| *Convolvulus arvensis* | 0.298 |
| *Ipomoea ternifolia* | 0.306 |
| *Ipomoea cordatotriloba* | 0.29 |
| *Ipomoea alba* | 0.291 |
| *Ipomoea hederacea* | 0.303 |
| *Ipomoea nil* | 0.285 |
